# Targeted long-read sequencing enriches disease-relevant genomic regions of interest to provide complete Mendelian disease diagnostics

**DOI:** 10.1172/jci.insight.183902

**Published:** 2024-09-12

**Authors:** Kenji Nakamichi, Jennifer Huey, Riccardo Sangermano, Emily M. Place, Kinga M. Bujakowska, Molly Marra, Lesley A. Everett, Paul Yang, Jennifer R. Chao, Russell N. Van Gelder, Debarshi Mustafi

**Affiliations:** 1 Department of Ophthalmology, University of Washington, Seattle, Washington, USA.; 2 Roger and Karalis Johnson Retina Center, Seattle, Washington, USA.; 3 Ocular Genomics Institute, Department of Ophthalmology, Massachusetts Eye and Ear, Harvard Medical School, Boston, Massachusetts, USA.; 4 Casey Eye Institute, Oregon Health & Science University, Portland, Oregon, USA.; 5 Departments of Laboratory Medicine and Pathology and Biological Structure, University of Washington, Seattle, Washington, USA.; 6 Brotman Baty Institute for Precision Medicine, Seattle, Washington, USA.; 7 Division of Ophthalmology, Seattle Children’s Hospital, Seattle, Washington, USA.

**Keywords:** Genetics, Ophthalmology, Genetic diseases, Molecular genetics, Retinopathy

## Abstract

Despite advances in sequencing technologies, a molecular diagnosis remains elusive in many patients with Mendelian disease. Current short-read clinical sequencing approaches cannot provide chromosomal phase information or epigenetic information without further sample processing, which is not routinely done and can result in an incomplete molecular diagnosis in patients. The ability to provide phased genetic and epigenetic information from a single sequencing run would improve the diagnostic rate of Mendelian conditions. Here, we describe targeted long-read sequencing of Mendelian disease genes (TaLon-SeqMD) using a real-time adaptive sequencing approach. Optimization of bioinformatic targeting enabled selective enrichment of multiple disease-causing regions of the human genome. Haplotype-resolved variant calling and simultaneous resolution of epigenetic base modification could be achieved in a single sequencing run. The TaLon-SeqMD approach was validated in a cohort of 18 individuals with previous genetic testing targeting 373 inherited retinal disease (IRD) genes, yielding the complete molecular diagnosis in each case. This approach was then applied in 2 IRD cases with inconclusive testing, which uncovered noncoding and structural variants that were difficult to characterize by standard short-read sequencing. Overall, these results demonstrate TaLon-SeqMD as an approach to provide rapid phased-variant calling to provide the molecular basis of Mendelian diseases.

## Abstract 2

We present a programmable, targeted long-read genome sequencing and epigenomic approach to fully characterize the genetic basis of Mendelian disease and better guide clinical management of disease.

## Introduction

The clinical heterogeneity of Mendelian disorders makes genetic testing essential in providing a precise diagnosis. However, despite remarkable advances in sequencing technologies over the past 20 years, nearly half of patients with Mendelian disease lack a complete molecular diagnosis (1–3). The precise identification of genotypic causes of disease as well as chromosomal phase information has taken on new importance, as treatment is only indicated for specific genetic defects in Mendelian conditions such as inherited retinal diseases (IRDs), for which FDA-approved gene therapy exists (4). The current standard–of-care testing approach to genetically diagnose diseased patients are targeted short-read exome-based sequencing panels (5). Compared with short-read exome sequencing, short-read genome sequencing (GS) provides increased diagnostic efficiency (6), but it has only provided a modest increase in molecular diagnosis (7). The missing genetic causality of disease is thought to reside in genomic regions of known disease-causing loci comprising structural (8) and noncoding variants (9, 10) of the genome, but may be difficult to sequence with short reads. More importantly, short-read GS methods do not yield haplotype information (11), which requires subsequent familial segregation studies to establish a molecular diagnosis in cases of autosomal recessive inheritance.

Long-read GS approaches from Pacific Biosciences and Oxford Nanopore Technologies (ONT) (12) have the potential to overcome these limitations by readily sequencing intronic and flanking genomic regions. Furthermore, by linking variants on single long-reads, long-read GS offers the added benefit of genomic phase information to provide a molecular diagnosis from the proband alone (13, 14). Whereas long-read GS offers immense genomic information, data storage and processing can make analysis costly and burdensome (15). In practice, Mendelian diseases are predominantly caused by diseased alleles located within a limited number of genomic loci, so focused genome-level sequencing of particular disease-causing loci would be more clinically relevant. Furthermore, this would eliminate the ethical issues and familial burden of managing incidental findings uncovered by GS unrelated to the diagnostic aim (16, 17), which can be a reason families defer genetic testing (18). However, current methods for targeted sequencing are labor intensive and not easily modifiable. Target panel enrichment with solution-based selection methods (19), commonly used in commercial exome-based gene panel testing, are difficult to modify to include new genomic regions. Genomic regions can be targeted with Cas9 to ligate adapters for long-read sequencing of multiple loci, but this strategy is limited by the size of fragments that can be targeted (20) and requires significant effort if targeting multiple loci.

To overcome these limitations, we leveraged long-read sequencing technology from ONT with a real-time bioinformatic adaptive sequencing functionality that allows rapid classification of the generated current signal to determine whether a DNA molecule should be sequenced or not (21, 22). In this work, we show that targeted long-read sequencing of Mendelian disease genes (TaLon-SeqMD) is customizable to multiple genomic loci (here, all IRD-associated genes). We developed metrics to analyze proper targeting of genomic loci to generate an optimized targeting genomic reference for use with standardly prepared genomic DNA (gDNA) libraries. After benchmarking the performance of TaLon-SeqMD in individuals who previously underwent CLIA-approved clinical molecular testing, we utilized TaLon-SeqMD to solve the genetic basis of disease in 2 individuals with a clinical presentation consistent with IRD, but with prior inconclusive genetic testing.

## Results

### Optimized genomic reference targeting provides focused depth of coverage for haplotype-resolved variant calling of targeted genomic loci.

With adaptive sampling on the ONT platform, emergent reads of single DNA molecules are compared in real time against a database of desired (positive selection) or undesired (negative selection) sequences, and unwanted sequences are aborted by reversal of charge at the level of individual nanopores. To evaluate the use of adaptive sampling to accurately identify IRD variants, we designed a custom panel encompassing a comprehensive list of genes implicated in IRDs (*n* = 373, Supplemental Table 1; supplemental material available online with this article; https://doi.org/10.1172/jci.insight.183902DS1). For each gene, the entire locus and flanking 50 kb of sequence in each direction were targeted via positive selection. A 50-kb flank was chosen so that long reads originating outside of each gene were captured and that entire gene was effectively covered. In total, the panel covered 54.3 megabases (Mb), corresponding to approximately 1.7% of the human haploid genome. For sequencing library preparation, high molecular weight genomic DNA was extracted from blood samples of consenting individuals for sequencing on ONT MinION flow cells. Real-time basecalling was carried out using the “super-accurate” model parameters on a custom Linux-based computing workstation equipped with 2 NVIDIA RTX A6000 graphics cards and AMD Treadripper Pro 4995WX 64-core, 128-thread desktop processor.

We first determined the optimal settings required to efficiently target the different genomic loci using the Genome Reference Consortium Human Build 38 (GRCh38) (23). A browser extensible data (BED) file of genomic coordinates from GRCh38 of each of the 373 IRD genomic regions was used for initial targeting. To assess proper targeting, the DNA bases expected to be mapped to each targeted genomic locus were calculated as a fraction of the total bases of all targeted loci (54.3 Mb). This was then compared to the observed DNA bases that were uniquely mapped to each targeted locus after a sequencing run. A linear regression of the observed versus expected bases revealed that 31 genomic loci exhibited lower than expected number of observed reads (Supplemental Figure 1A). Closer examination of these genomic regions revealed that entire genes or portions of a gene did not map correctly due to inherent errors in the GRCh38 assembly (Supplemental Figure 1, B and C). Masking these selected genomic regions and generating a new GRCh38 reference assembly file was a major advance that led to proper targeting and improved correlation of these points on the linear regression (Supplemental Figure 1, D–F). Moreover, this method of read alignment assessment for a sequencing run can be modified for any targeted set of genomic loci to determine the optimal targeting reference necessary for accurate variant calling.

**Figure 1:**
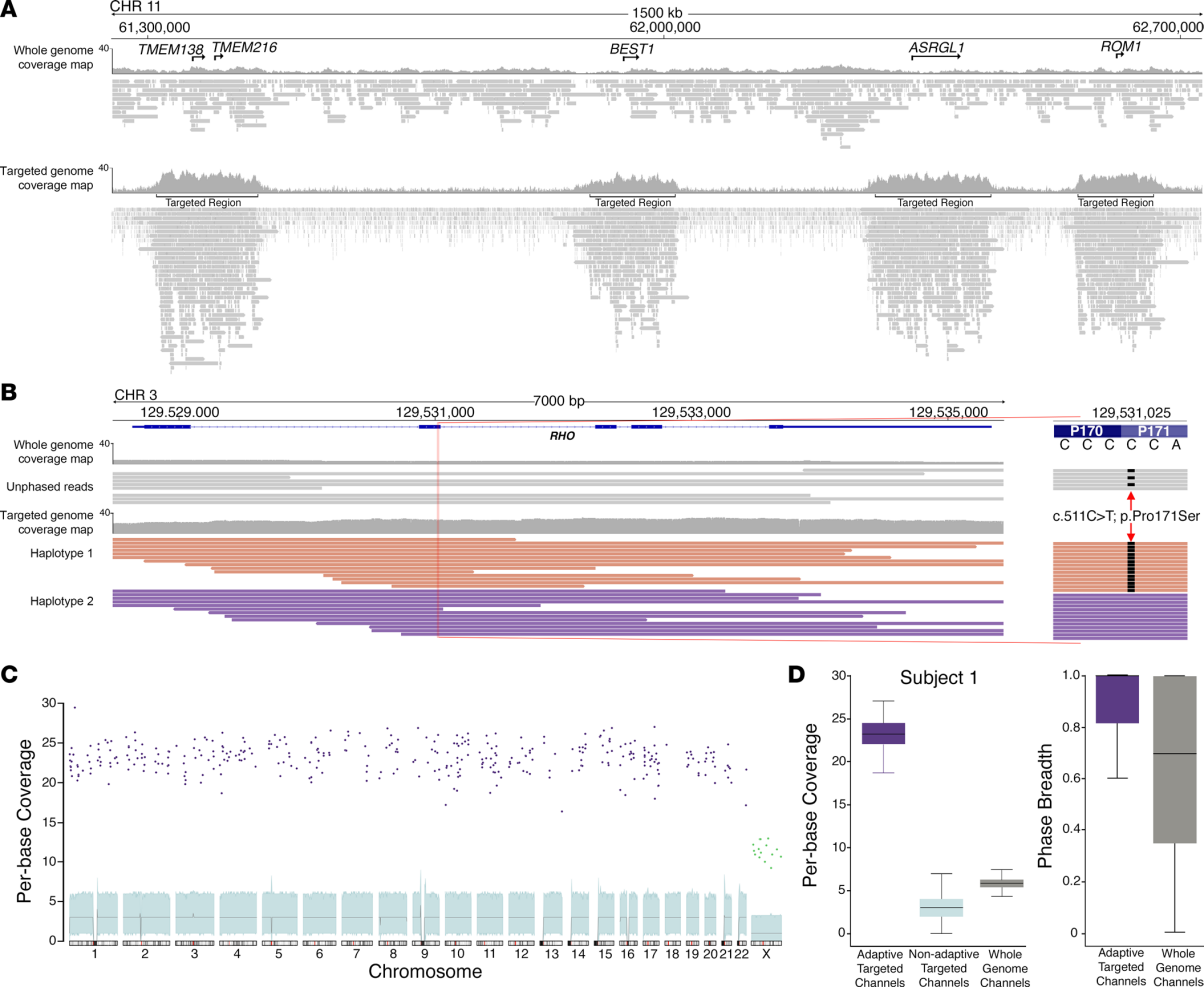
TaLon-SeqMD generates selective whole-gene coverage of IRD genes to allow phased-variant identification. (**A**) Coverage maps and sequencing alignments of a 1500-kb region of chromosome 11 with whole-genome sequencing (WGS) and targeted sequencing of IRD disease-gene loci in that region (*TMEM138*, *TMEM216*, *BEST1*, *ASRGL1*, *ROM1*) demonstrate that bioinformatic targeting provides focused depth of sequencing. The locations of the targeted regions are marked. (**B**) The rhodopsin (*RHO*) locus is shown to demonstrate the increased depth obtained from a targeted run compared with a whole-genome run allows for haplotyping to conclusively demonstrate that a disease variant segregates on a single allele. (**C**) Examination of the coverage across the genome shows selected enrichment of bases covered by the panel genes (blue dots) compared with background coverage of the genome from nonadaptive reads. (**D**) Box-and-whisker plots show that targeted panel sequencing results in 25× mean per-base coverage compared with 3× with nonadaptive reads and 5× with WGS on a single MinION flow cell. Calculation of phase breath of the data revealed that TaLon-SeqMD was able to phase significantly more of the targeted genomic regions than WGS.

With proper targeting parameters established and optimized for our IRD panel, we sought to compare sequencing efficiency of TaLon-SeqMD to nontargeted long-read GS using DNA libraries prepared from the same individual with IRD (subject 1) on ONT MinION flow cells. In the first flow cell we carried out long-read GS without any targeting, and in the second flow cell we utilized adaptive sampling to target the preselected 373 IRD genomic loci using our updated reference file. TaLon-SeqMD produced enhanced depth of coverage of all 373 loci, whereas with whole-genome sequencing there were gaps, with regions of interest exhibiting little to no read coverage (Figure 1A and Supplemental Figure 2). The depth of sequencing allowed for phasing of the disease-causing variant in the rhodopsin (*RHO*) gene in this individual with the TaLon-SeqMD run, but not with the GS run (Figure 1B). The mean per-base coverage of the adaptive sampling channels was 25× compared with 3× from the nonadaptive sampling channels (Figure 1C), whereas the GS flow cell resulted in a modest 5× mean per-base coverage of IRD gene loci (Figure 1D). This reduced depth of sequencing resulted in a statistically significant decrease in phased regions of the targeted loci with GS. TaLon-SeqMD resulted in phasing of 85% of targeted loci (median of 100%) compared with 64% (median of 69%) with GS (Figure 1D). More importantly, there were entire genomic regions that were unable to be phased with the GS run.

### TaLon-SeqMD validates clinical sequencing data and provides full molecular diagnoses in genotypically diverse Mendelian disease cases.

**Figure 2:**
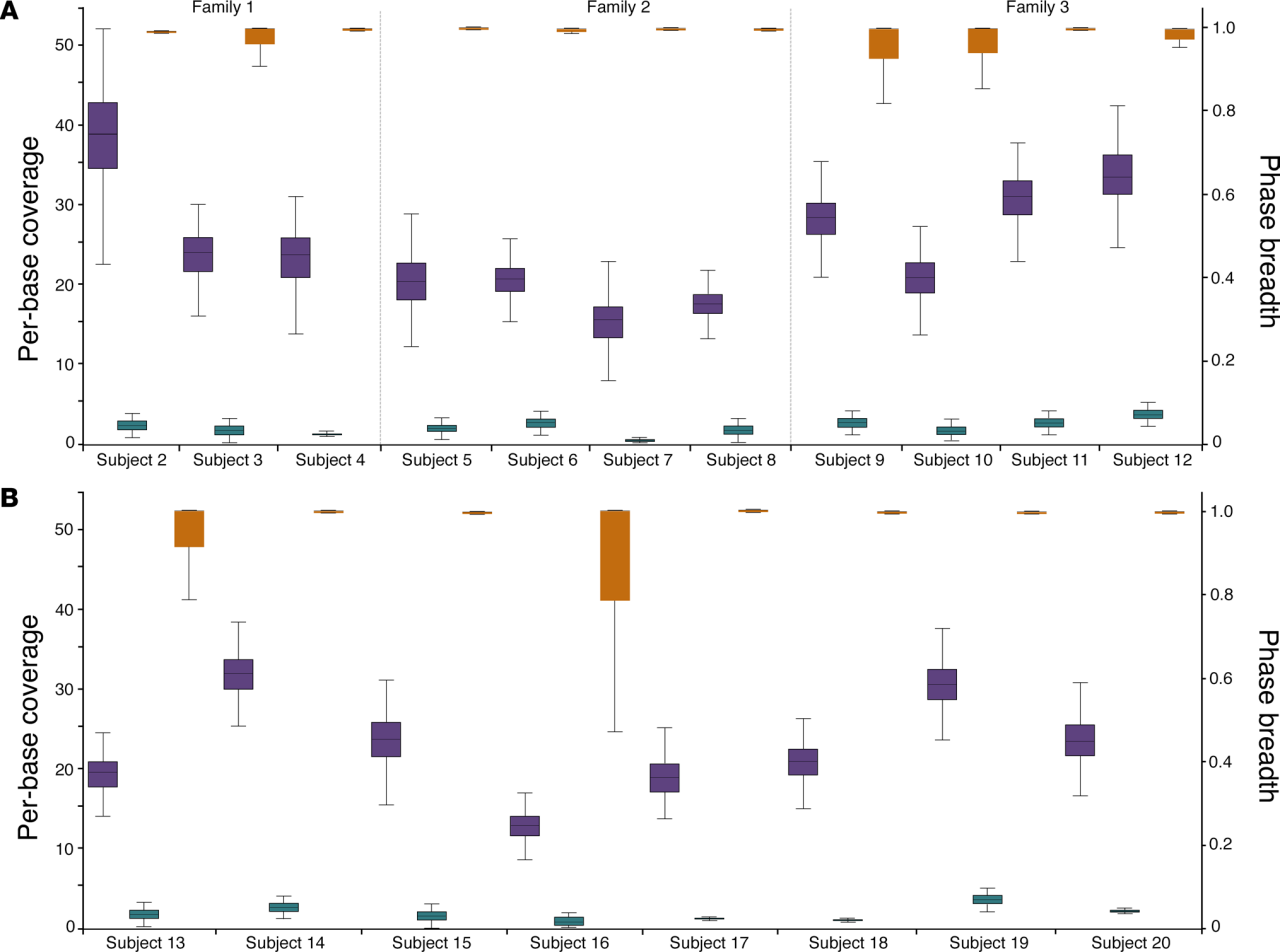
TaLon-SeqMD across a cohort of familial and isolated proband samples provides selective enrichment of targeted genomic loci and phasing of disease-relevant genes to allow for a full molecular diagnosis in each case. Box-and-whisker plots illustrate the depth of coverage from adaptive targeted channels (purple) and nonadaptive channels (green) as well as the phase breadth (orange) for each subject. (**A**) Familial individuals were first assessed across 3 different families. TaLon-SeqMD demonstrated an average of 19-fold enrichment of targeted genomic loci and average phase breadth of 0.91, allowing complete verification of allelic architecture of disease variants. (**B**) Eight proband samples were then examined with TaLon-SeqMD and 16-fold mean enrichment of targeted genomic loci and average phase breadth of 0.91 was achieved across samples. For subject 16 who exhibited the lowest overall sequencing output that led to a mean phase breadth of 0.84, the clinically relevant variants could still be phased to reassign a VUS to provide a full molecular diagnosis.

To establish that TaLon-SeqMD can provide diagnostic information in Mendelian disease cases, we enrolled individuals that had undergone clinical molecular testing in CLIA-approved facilities. We analyzed DNA samples from 19 additional individuals, which included 14 affected IRD individuals and 5 unaffected family members. Across all samples, the mean per-base coverage of the 373 loci was 22.74 ± 2.88, with a mean read length of 7090 ± 2595 bases from the adaptive sampling channels. We achieved greater than 15-fold enrichment on average across all samples of our 373 targeted genomic loci (Figure 2). More importantly, we demonstrated that greater than 91% (0.91 ± 0.04) of all targeted genomic loci were fully phased across all samples from a single sequencing run. The median across all samples was 100%, and examination of the lower quartile revealed that greater than 96% of targeted genomic loci were fully phased across the samples. Most importantly, despite the range in panel coverage, read length, and phase breadth across the cohort, we were able to deliver a molecular diagnosis in each case.

We initially examined familial data to verify phased variants in disease-affected individuals (Figure 2A). We carried out TaLon-SeqMD on 3 families with disease variants in *USH2A* (subjects 2–4) *TPP1* (subjects 5–8), and *USH2A* (subjects 9–12). In each case we were able to correctly phase the proband samples with our approach and confirm the allelic architecture with familial data. We then shifted our attention to 8 disease-affected individuals in whom variant phasing was not possible due to lack of familial DNA (Figure 2B). We first showed how different arrangements of complex variants in *ABCA4* can lead to varied phenotypic presentations in 2 cases (subjects 13 and 14). The ability to phase variants allowed reclassification variants of uncertain significance (VUS) to likely pathogenic to provide a complete molecular diagnosis in 2 cases (subjects 15 and 16). In 1 case (subject 17) without clinical testing results at the time of TaLon-SeqMD, we showed that pathogenic variants lying over 526 kb apart could be identified and phased to provide a rapid molecular diagnosis. We further show that in 2 cases (subjects 18 and 19) that TaLon-Seq provided a full molecular diagnosis after indeterminate clinical short-read sequencing. Finally, we show in subject 20 that the ability to sequence native DNA allows decoding the base methylation signal to identify potentially important epigenetic features of the genome in the context of disease.

### Allelic architecture of variants revealed by TaLon-SeqMD can prioritize variants for further analysis to establish a molecular diagnosis.

**Figure 3:**
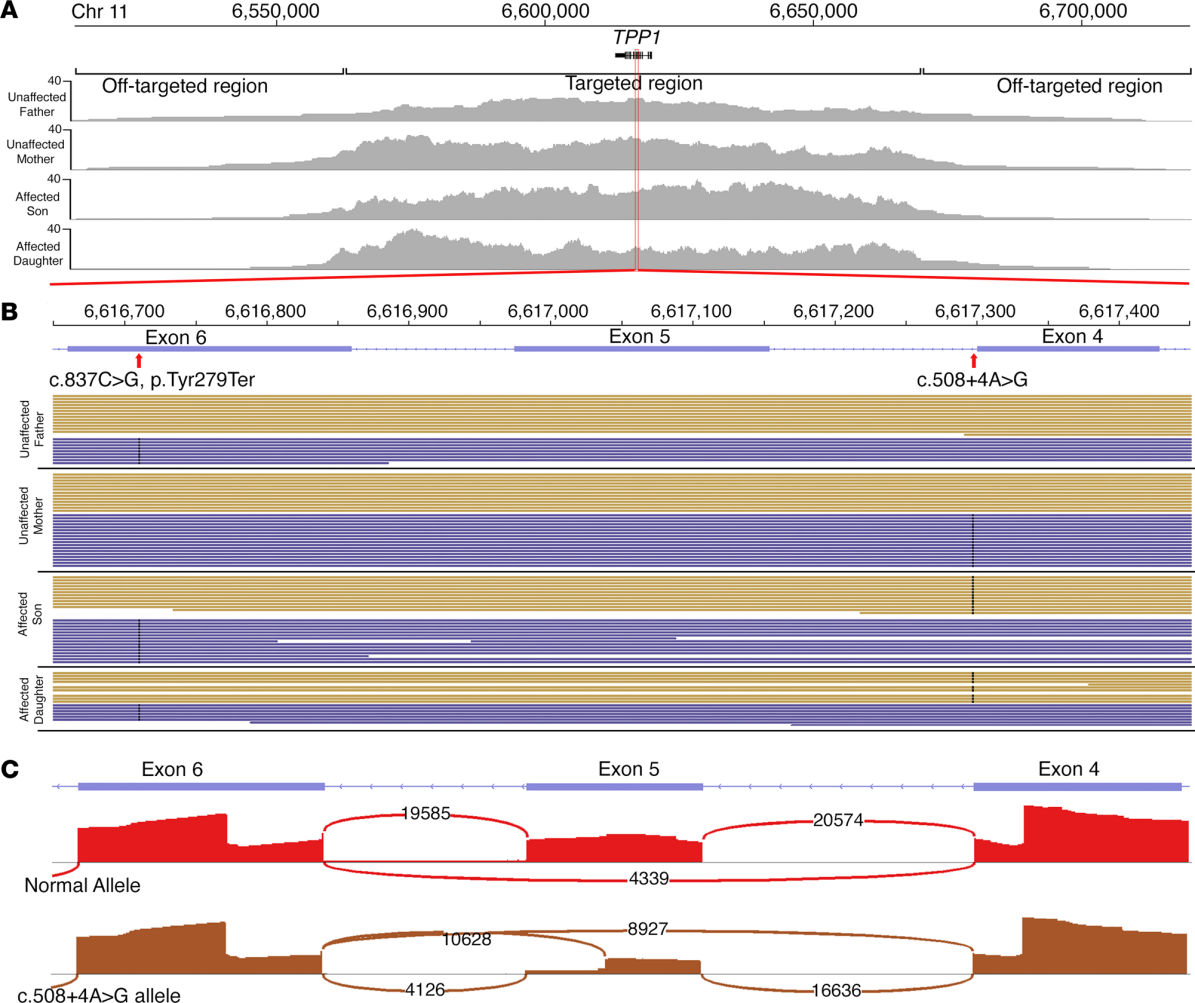
Targeted panel sequencing reveals haplotagged variants in the *TPP1* gene. (**A**) Coverage maps show the sequence alignments at the *TPP1* locus and surrounding 50-kb region denoted by on-target reads. There is negligible coverage in off-target regions flanking the gene. (**B**) Closer view of the regions encompassing exons 4 to 6 reveal the location of the 2 potential disease-causing SNVs. The unaffected parents are shown to each possess 1 variant, whereas both affected children possess both. (**C**) Since both of the affected individuals had atypical forms of Batten disease, the intronic variant was hypothesized to be a hypomorph, which was demonstrated to display variant-induced aberrant splicing compared with the normal allele, which is denoted graphically with a sashimi plot.

DNA in each prepared library is stochastically sampled to perform positive selection for full-length sequencing, so we hypothesized that expansion from a single gene to 373 genomic loci should not affect depth of coverage. To this hypothesis, we first examined familial data (family 1) of 2 affected siblings (subjects 2 and 3) with Usher syndrome type 2 (USH2) and their unaffected mother (subject 4) in whom we had previously carried out targeted long-read single-gene analysis of *USH2A* (13). We found that expanding our targeting to 373 genomic loci did not result in decreased coverage of *USH2A* relative to single-gene-targeting sequencing (Supplemental Figure 3) and could still provide phased-variant calling for molecular diagnosis. We next examined family 2 afflicted with a syndromic IRD caused by variants in the *TPP1* gene, which is one of the most prevalent forms of juvenile neuronal ceroid lipofuscinosis (JNCL) (24), to better understand how allelic architecture may influence disease phenotype. Clinical exome testing had identified a nonsense variant (c.837C>G, p.Tyr279Ter) and a potential second variant in a noncoding region (c.508+4T>C) in both affected siblings. Targeted variant testing of unaffected parents (subjects 5 and 6) revealed that they each harbored 1 of the 2 variants, providing evidence that the suspected disease variants lie in *trans*. Phenotypically, the older sibling (subject 7) had evidence of retinal disease, but the younger sibling (subject 8) had normal retinal findings. TaLon-SeqMD of all 4 family members provided on average 20× coverage of all targeted loci and phasing of over 90% of all targeted genes, including full-phased coverage of *TPP1* (Figure 3A). Closer examination of the 800-bp region of *TPP1* containing exons 4–6 demonstrated that the unaffected parents each harbored 1 variant, whereas both affected siblings harbored both the nonsense and noncoding variants in a *trans* configuration (Figure 3B), confirming previous clinical testing data. Our analysis pipeline identified the nonsense variant in *TPP1* as the top disease-variant candidate. Moreover, since we had the benefit of also sequencing other syndromic retinal disease loci, we were able to rule out other genetic etiologies. The atypical presentation of JCNL suggested that the allele harboring the noncoding variant likely retained some activity due to reduced, but not abolished, protein product (25). To test this hypothesis, we carried out a splicing assay that showed the noncoding allele, c.508+4A>G, functions as a nonessential splice site leading to aberrant splicing compared with the normal allele, which led to decreased, but not complete, abolishment of the native protein product (Figure 3C), thereby providing a biochemical basis for a molecular diagnosis of atypical *TPP1*-associated JNCL.

### TaLon-SeqMD provides phased-variant identification for allelic localization of complex variants to explain phenotypic findings and aid in VUS reassignment.

**Figure 4:**
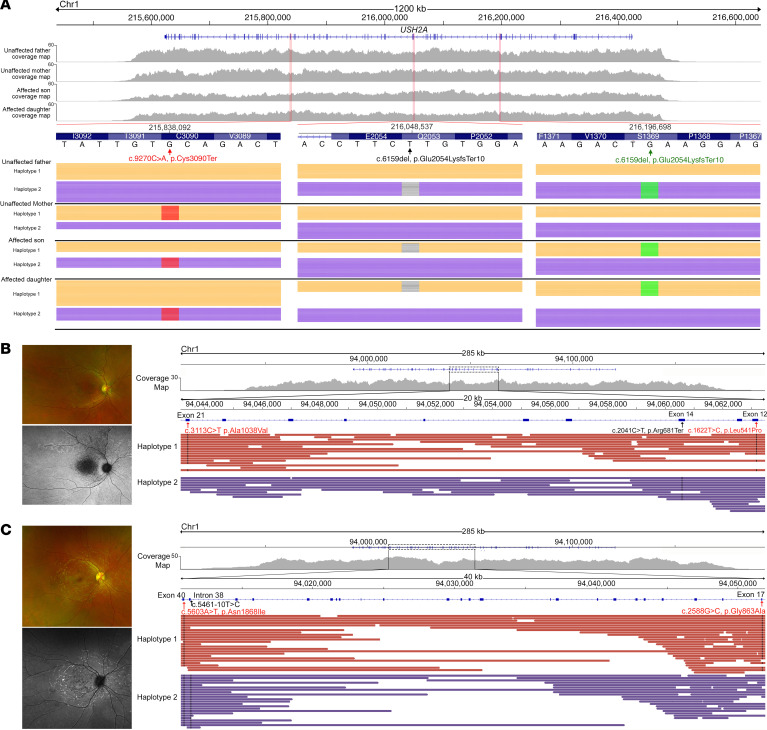
Haplotype-resolved assembly of complex phased variants provides insight into differential disease phenotypes. (**A**) Two affected siblings with *USH2A*-associated Usher syndrome were found to have 3 pathogenic variants in *USH2A* from clinical testing without phase information. Targeted long-read sequencing demonstrated that the unaffected father harbored 2 pathogenic variants in *cis*, whereas the unaffected mother harbored 1 pathogenic variant. Targeted long-read GS correctly identified the variant architecture from the probands alone with the 2 variants inherited from the father in *trans* to the variant inherited from the mother. Variant architecture can influence disease progression, as evidenced in *ABCA4*-associated Stargardt disease. (**B**) An adolescent subject with severe disease, as exhibited by chorioretinal atrophy with a large region of hypoautofluorescence in the central macula, had 3 pathogenic alleles, 2 of which were severe and were in a *trans* configuration. (**C**) In comparison, a middle-aged individual with mild disease, as exhibited by hyperautofluorescent flecks without atrophy in the central macula, also had 3 pathogenic alleles, but had 2 hypomorphic alleles in *cis*, both of which were in *trans* to a severe allele.

Complex alleles, where 2 or more disease variants may lie in *cis*, can complicate disease diagnosis. Understanding of the precise allelic architecture of disease variants can be critical in disease prognosis. To investigate these cases, we describe family 3 in which 2 affected siblings with USH2 were found to harbor 3 pathogenic variants in the *USH2A* gene with clinical exome panel testing. Targeted variant testing of the unaffected parents revealed that the father harbored 2 of the variants, whereas the mother harbored the other variant. TaLon-SeqMD of all 4 family members (subjects 9–12) produced fully phased coverage of the large *USH2A* locus, spanning 1.2 Mb, across all 4 individuals. The data demonstrated that the father harbored 2 heterozygous disease-causing variants in *cis* (c.6159del and c.4106C>T) and the mother harbored 1 heterozygous disease-causing variant (c.9270C>A). Both affected offspring exhibited all 3 variants, with the c.6159del and c.4106C>T variants in *cis* and the c.9270C>A variant in *trans* to the other 2 (Figure 4A). Despite the c.4106C>T and c.9270C>A variants being over 614 kb apart, TaLon-SeqMD was able to phase the variants from a single sequencing run. Whereas all 4 family members underwent long-read sequencing, these results demonstrate that the precise allelic architecture of the complex disease variants could be revealed from sequencing of a single individual without the need for familial samples.

The allelic arrangement of disease variants has been shown to be integral in determining the prognosis of particular Mendelian diseases such as *ABCA4*-related Stargardt disease (26). This gene has well characterized hypomorphic variants that can lead to different disease phenotypes (27, 28). We present 2 cases in which familial DNA was not available to determine the allelic architecture and thus explain phenotypic differences in *ABCA4*-related Stargardt disease. In the first case (subject 13), TaLon-SeqMD revealed that a hypomorphic variant (c.3113C>T, p.Ala1038Val) was in *cis* with a severe disease-causing variant (c.1622T>C, p.Leu541Pro), both of which lie in *trans* to another severe variant (c.2041C>T, p.Arg681Ter). This individual exhibited early-onset vision loss in adolescence with severe clinical phenotype noted on retinal imaging (Figure 4B). In the second case (subject 14), TaLon-SeqMD revealed that 2 hypomorphic variants (c.5603A>T, p.Asn1868Ile and c.2588G>C, p.Gly863Ala) lie in *cis* and are in *trans* to a severe variant (c.5461-10T>C). Molecular studies have shown that when the hypomorphic variants c.5603A>T/p.Asn1868Ile and p.2588G>C/p.Gly863Ala lie in *cis* there is relatively normal protein expression and functionality (29). This is consistent with a later onset of disease, as exhibited in this individual who only had mild retinal findings and preserved visual functionality by her mid 30s (Figure 4C).

This demonstrates that in the absence of familial DNA, TaLon-SeqMD can provide precise variant-level insight from the allelic arrangement revealed from phased data sets. In certain cases, demonstrating that the VUS lies in *trans* to a known pathogenic variant is the final criterion needed to reassign the pathogenicity (30) and provide a full molecular diagnosis. We show this in an adolescent with early-onset *ABCA4*-related Stargardt disease (subject 15) who harbors the known pathogenic variant c.5461-10T>C noted in the previous individual (Figure 3C). TaLon-SeqMD demonstrated that the previously identified VUS from clinical sequencing, c.3413T>C, p.Leu1138Pro, was in *trans* and allowed reassignment to likely pathogenic (Supplemental Figure 4). Similarly, deducing the allele-level variant architecture in an individual (subject 16) with *PDE6A*-associated retinitis pigmentosa (RP) allowed reassignment of c.1646T>C, p.Leu549Pro to likely pathogenic (Supplemental Figure 5).

### Phased-variant calls from TaLon-SeqMD provide rapid disease diagnostics in autosomal recessive cases of disease.

**Figure 5:**
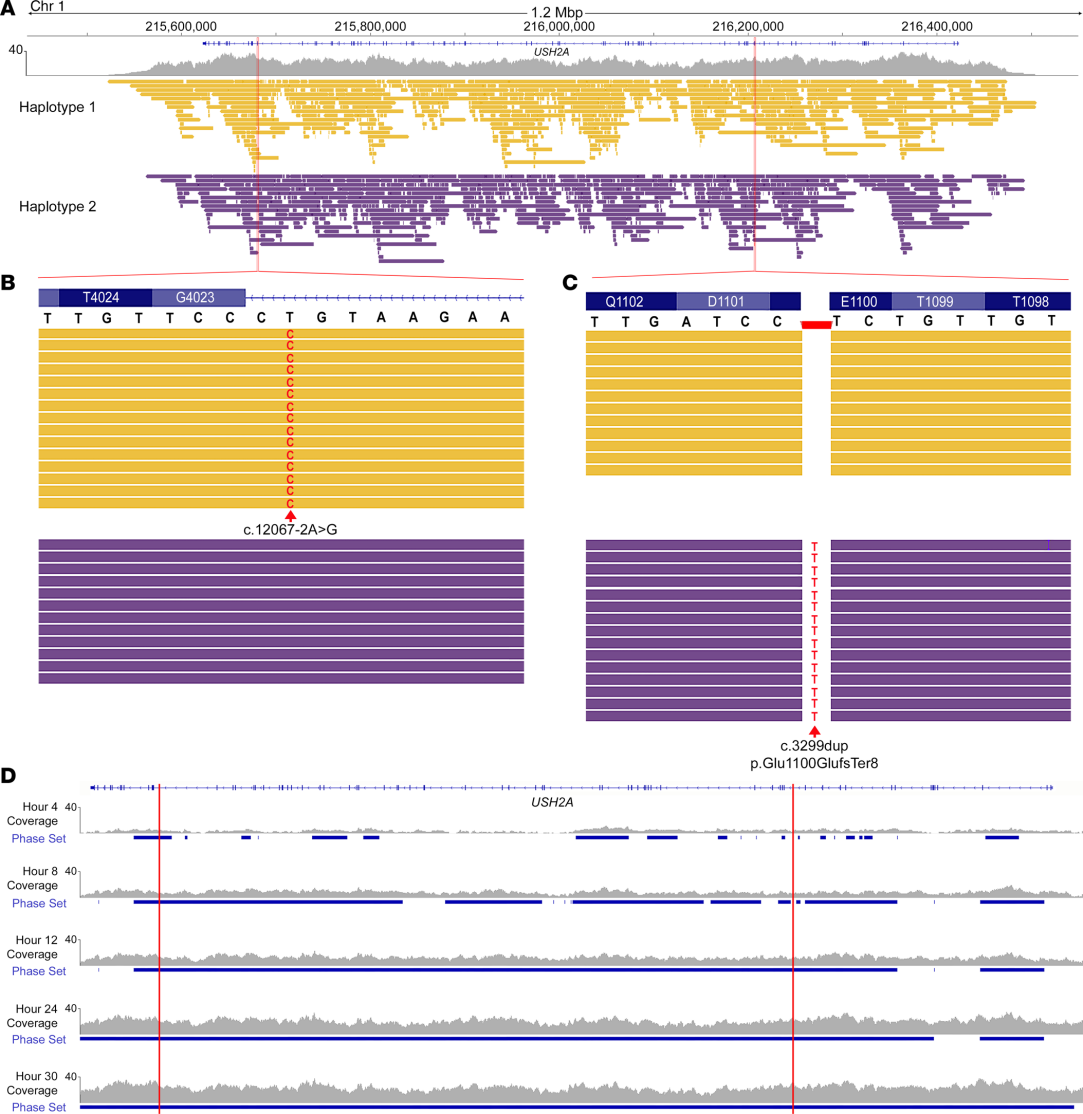
Targeted panel genome sequencing allows for rapid identification of disease-causing variants. (**A**) In an affected individual with no prior genetic testing, targeted long-read panel sequencing revealed 2 likely disease-causing variants in the *USH2A* gene, (**B**) an SNV noted to be pathogenic, and (**C**) a duplication leading to a frameshift and early termination that was in *trans*. (**D**) Post hoc analysis of the sequencing data revealed that the 2 variants in *USH2A* were identified and properly phased within 12 hours of sequencing, whereas the entire *USH2A* gene could be phased 30 hours after sequencing.

A critical issue in Mendelian disease diagnostics is the turnaround time for clinical results, which can therefore impact treatment options. Furthermore, initial genetic testing results are often incomplete in autosomal recessive diseases since the chromosomal phase information is not available. Thus, secondary analysis must then be carried out, which extends the time for complete diagnosis, and which can only occur if familial DNA is available for analysis. We show that TaLon-SeqMD not only provides phased genomic data sets, but that it does so in a rapid timeline using a single MinION flow cell. We enrolled an individual who had just been clinically diagnosed with RP and had a history of congenital hearing loss, which was strongly suggestive of a syndromic disorder such as USH2. The individual (subject 17) was having a sample sent for clinical genetic testing for an IRD so we simultaneously obtained a sample to carry out TaLon-SeqMD. After sequencing and analysis, we found that the individual exhibited a known pathogenic splicing variant in the *USH2A* gene (c.12067-2A>G) along with a previously uncharacterized frameshift variant (c.3299dup, p.Glu1100GlufsTer8) in *trans*, which was predicted to be pathogenic (Figure 5, A–C). We carried out post hoc analysis of the data to identify when sufficient reads were present to phase the disease variants and found that within 12 hours of sequencing, the 2 variants residing over 526 kb apart had been identified and phased (Figure 5D). Clinical genetic test results were available after 7 weeks, confirming both variants in *USH2A* found from TaLon-SeqMD, but without the benefit of phase information.

### Comprehensive genomic profiling with TaLon-SeqMD can provide full molecular diagnosis in cases with missing heritability after clinical sequencing.

**Figure 6:**
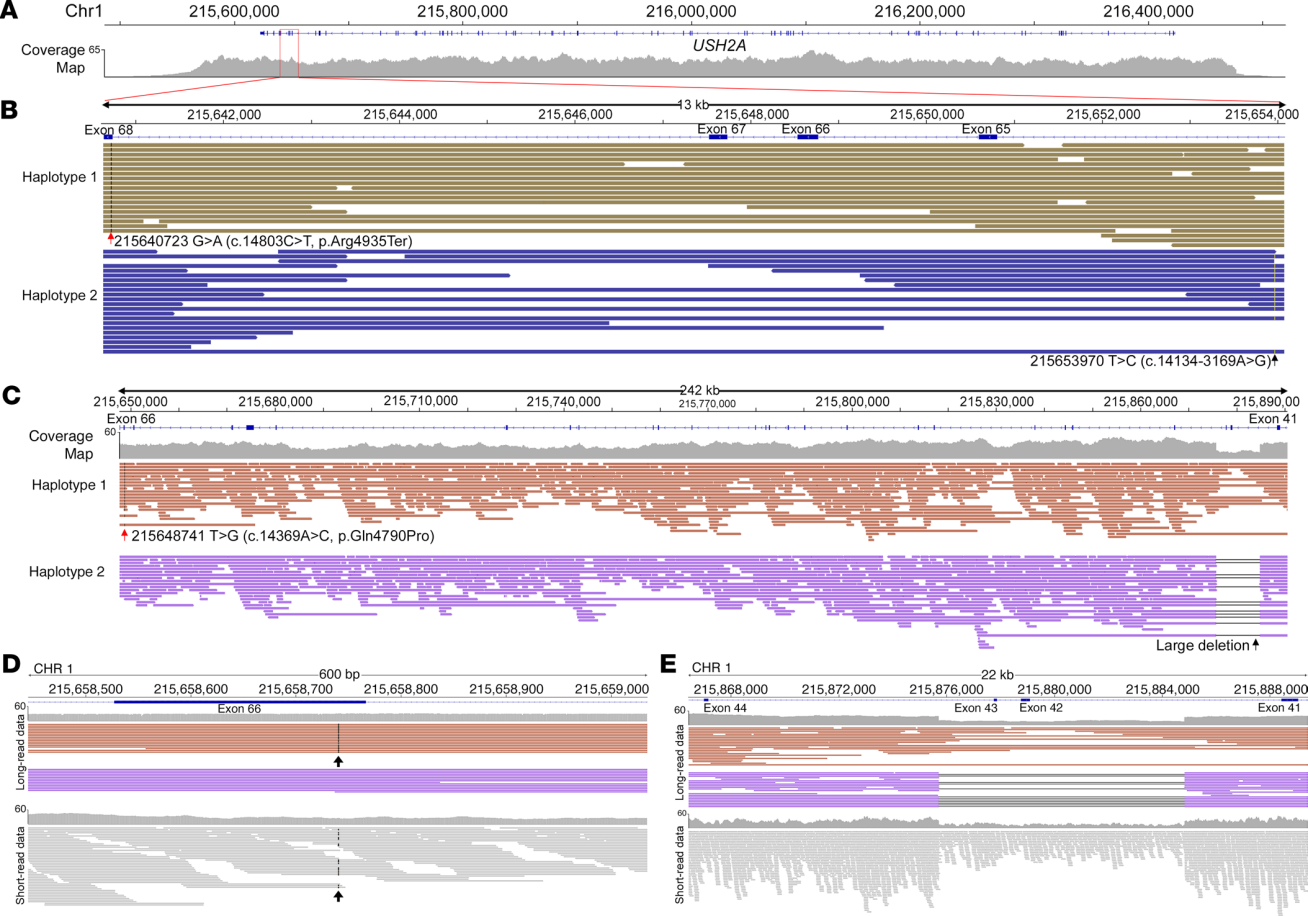
Targeted whole-genome long-read sequencing can detect complex SVs and deep intronic variants to provide insight in cases of missing heritability. In 2 cases of individuals with Usher syndrome, a pathogenic coding SNV was found with initial clinical exome-based panel testing. (**A**) The complete *USH2A* locus was covered, which allowed examination of noncoding regions. (**B**) Closer view of a 13-kb region encompassing intron 64 to exon 68 shows the known coding variant in exon 68 (red arrow) and the noncoding variant (black arrow) lie in *trans*. (**C**) In the second case, we show the 242-kb region encompassing the known coding variant and the large structural deletion encompassing exons 42 and 43, with the 2 variants segregating in *trans*. (**D**) Closer examination of the coding SNV shows long-read data are able to segregate the variant on a single haplotype. (**E**) Long-read data of the SV are able to again show it segregates on a single chromosome, with precise breakpoint detection compared with short-read data.

We show that TaLon-SeqMD can be instrumental in monoallelic cases where only one pathogenic variant was identified after clinical exome-based sequencing. Not only can the second causative variant be identified with targeted long-read sequencing, but phased data can demonstrate that the second variant is in *trans* to the identified variant to further validate its role in disease. We show that phased data sets can provide complete molecular diagnosis in 2 autosomal recessive cases of USH2. In the first case a known pathogenic coding variant in *USH2A* had been identified with exome-based panel clinical sequencing. Targeted long-read sequencing of this individual (subject 18) identified a pathogenic noncoding deep intronic variant (c.141314-3169A>G) that leads to new pseudoexon activation leading to a premature termination codon (31). This variant was found to reside in *trans* to the previously identified coding variant (Figure 6, A and B), which provided a complete molecular diagnosis in this individual.

Another cause of missing heritability in monoallelic cases can be attributed to structural variants (SVs). SVs account for a lower percentage of IRD cases than that of single nucleotide changes and small insertions and deletions (8). This may be because SVs cannot be detected as reliably using standard short-read sequencing approaches. Long-read sequencing is superior in SV detection (32) and can thus better aid in the diagnosis of SVs contributing to IRDs (33). Furthermore, the higher resolution of sequences with long reads allows for more accurate SV detection to determine the precise breakpoint locations (34). We present a case (subject 19) where a pathogenic coding variant in *USH2A* was identified, but initial clinical exome panel testing did not identify a second variant. Targeted long-read sequencing showed that in addition to the known pathogenic variant, there was a large deletion encompassing exons 42 and 43 that resided in *trans* (Figure 6C). We carried out short-read genome sequencing as well in this individual to compare the 2 methods in identifying the SV. When examining the coding variant in exon 66, both approaches identified it, but long-read sequencing was able to provide phase information (Figure 6D). When examining the short-read genome data in the region of the SV, there is clear copy number variation suggestive of a deletion, but since reads do not span this region, it is unclear where the precise genomic breakpoints are (Figure 6E). We utilized a deep learning–based SV tool (35) to identify the exact breakpoints in our long-read data (Chr1: 215,875,713–215,884,830). More importantly, with the benefit of phasing the reads, we demonstrate that the SV lies in *trans* to the coding variant and is the likely cause of a disease in this individual.

### Native DNA sequencing provides DNA base methylation information to localize disease-relevant epigenetic signals.

**Figure 7:**
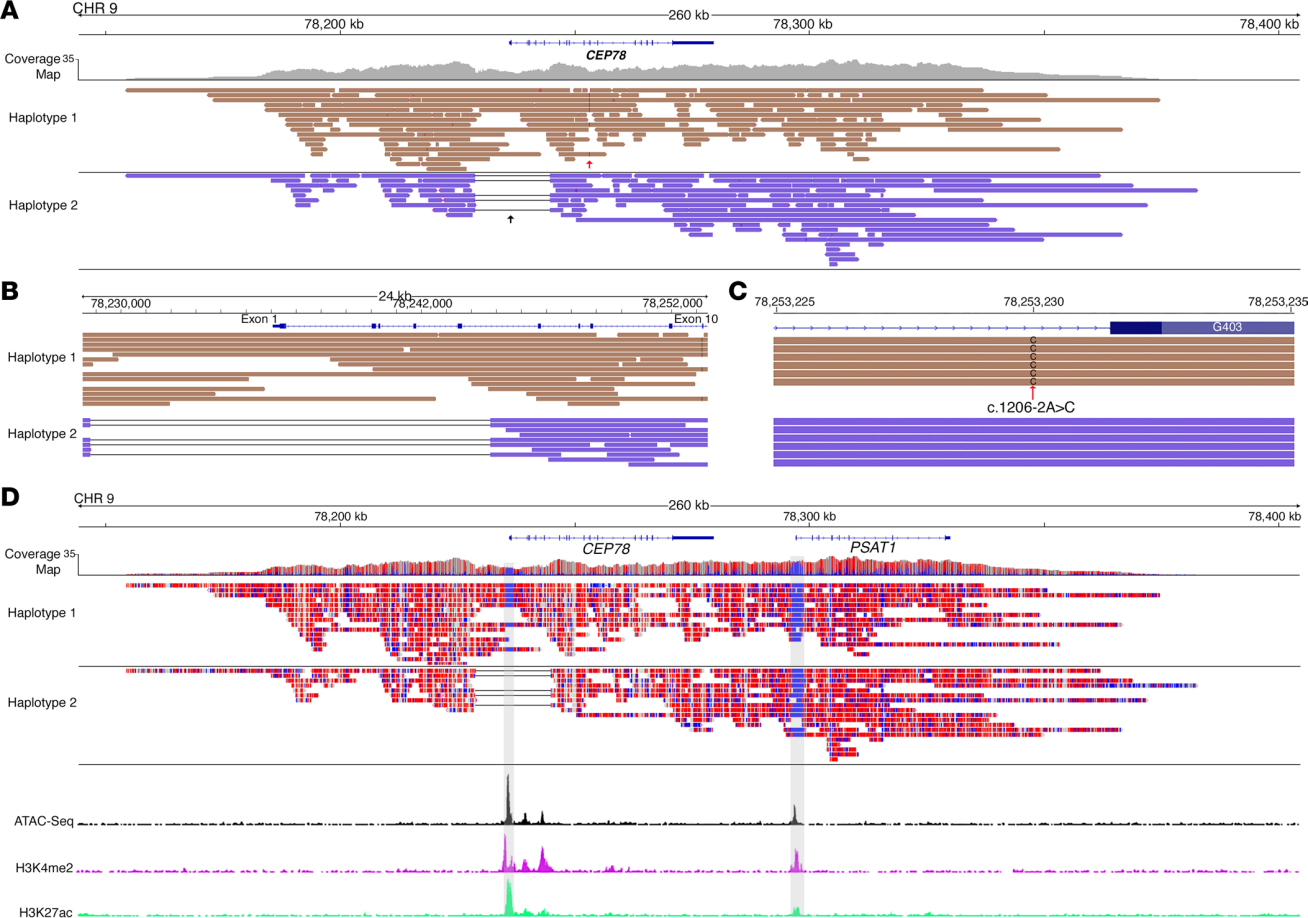
Sequencing of native DNA allows decoding of methylation data that correlates with transcriptionally active regions of the genome. (**A**) Targeted long-read genome sequencing of an individual with *CEP78*-associated IRD demonstrated full genomic coverage of the disease locus and elucidation that clinically identified (**B**) SV and (**C**) SNV resided in *trans*. Closer examination of the SVs showed that the precise breakpoints not only encompassed exons 1–5, but also upstream of the gene. (**D**) Base methylation analysis was carried out, and there were clustered regions of hypomethylation (in blue) that correlated well with measures of chromatin accessibility (ATAC-seq) and active histone activity (H3Kme2 and H3K27ac). Examination upstream of the *CEP78* gene showed that the other region of hypomethylation corresponded to similar chromatin measures in the *PSAT1* gene.

Another advantage of TaLon-SeqMD is that base modifications can be captured with a standard run since native DNA is sequenced. This allows epigenetic profiling, which is important since *cis*-regulatory elements (CREs) can harbor disease-causing variants (36). We present a case (subject 20) with early-onset pericentral rod-cone dystrophy who had clinical sequencing that identified 2 pathogenic variants, 1 coding a single nucleotide variant (SNV) and 1 SV, in the *CEP78* gene (Figure 7A). This individual was adopted, and since familial DNA was not available for variant segregation, we carried out TaLon-SeqMD to phase the variants. Clinical sequencing had indicated that exons 1–5 were deleted, but examination of the precise breakpoints from our long-read data (chr9: 78,228,783–78,244,408) demonstrated that the deletion started upstream of exon 1 of *CEP78* (Figure 7B) and was in *trans* to the SNV (Figure 7C). Since ONT sequencing relies on electric current intensity to assign base reads, it can detect DNA modifications that exist between the output electrical signal of modified and unmodified bases. More importantly, since this can be implemented as part of a normal sequencing run, we are able to examine the output DNA base methylation data without any special sample processing. In this case, we examined the 5-methylcytosine (5mc) data and found there was a focal region of hypomethylation clustered in the promoter region of *CEP78*, which the SV encompassed. When we examined chromatin accessibility from assay for transposase-accessible chromatin sequencing (ATAC-seq) data along with markers of active CREs such as acetylated lysine 27 on histone 3 (H3K27ac) and the histone mark H3K4me2, which are epigenetic mark of enhancers and promoters, from retinal tissue (37), we found that these peaks perfectly correlated with this cluster of hypomethylated bases in *CEP78* (Figure 7D).

## Discussion

We present a programmable, targeted long-read genome sequencing and epigenomic approach and demonstrate the utility of this approach, termed TaLon-SeqMD, for fully characterizing the genetic basis of disease in a Mendelian condition. Since sequences of biological interest comprise only a small fraction of the human genome in Mendelian disease, the focused depth of sequencing with our targeted approach not only validated prior clinical testing, but haplotype phasing provided a complete molecular diagnosis in each case. For IRDs, as with most Mendelian disorders, the most common inheritance pattern is autosomal recessive. This approach can phase variants relative to known disease-causing variants, which allowed us to provide diagnostic variant identification in complex cases that had evaded characterization by standard clinical testing diagnostics. We also demonstrated that the longer read lengths provided by ONT can better identify large SVs since there is no drop-off in read quality that can limit short-read approaches. Moreover, since native DNA was sequenced, epigenetic signatures such as methylation signal could be obtained for each run to provide a surrogate of chromatin accessibility and active CREs of disease-causing loci.

A unique capability of this approach is that since sequencing effort is focused on disease regions of interest, coverage and phasing can be achieved on a more rapid time scale than clinical sequencing approaches. This is critical in Mendelian conditions such as in-born errors of metabolism, where genetic diagnosis can impact clinical management. We show in one case that within 12 hours of a targeted sequencing run, data had been generated to not only identify 2 disease variants, but also phase them to demonstrate the variants lie in *trans*. Comparatively, standard clinical exome panel data results were available after 7 weeks, albeit without phasing of the disease variants, thereby providing incomplete information compared with our approach. Long-read sequencing on the ONT platform has previously been shown to offer rapid identification of disease-causing variants in clinical settings (38, 39) on the time course of hours, similar to what we demonstrate in this study. However, these other approaches required significant cost in terms of consumables and computational resources. We were able to achieve similar results using a single MinION flow cell and more limited computational resources since only a fraction of the genome was targeted and analyzed, making TaLon-SeqMD accessible to individual clinical labs in the future. Furthermore, we demonstrate that a proper genomic reference must be generated to achieve accurate targeting of genomic regions of interest. Adaptive sampling uses genomic coordinates from an input human reference genome assembly provided by the user to achieve accurate targeting and sequencing selection; therefore, inaccurate genomic maps can influence sequencing parameters, which will be an important consideration as this approach is expanded to other Mendelian diseases. Finally, for optimal implementation of this approach the available computer hardware is critical. Adaptive sample rejection time, which directly correlates to enrichment levels, is influenced by computing resources for optimal assay performance. We were able to achieve on average 22× coverage of the targeted genomic regions across our cohort of 20 individuals using the MinION device, which can be further optimized in the future with more rapid sequence classification algorithms (40) to allow faster rejection times and thus even more focused sequencing depth and rapid variant identification from a single sequencing run.

Since GS provides the same sensitivity and accuracy in variant calling with lower average coverage compared with exome sequencing (41), the more modest depths attainable from a single TaLon-SeqMD run demonstrated in this work can provide clinical diagnostic information. More importantly, the ability to generate haplotype-resolved reads can narrow down potential disease-causing variants in monoallelic cases where disease-variant discovery can be focused on a single haplotype. We show in autosomal recessive monoallelic cases that this allowed us to focus on the second haplotype to identify the causative variant in *trans*. We also show that native DNA sequencing provides us base modification data as well, which can be instructive of epigenetic signatures as we show with methylation signal overlap with known genomic markers of transcription and histone modification. Moreover, by sampling numerous disease-causing loci, this approach could identify potential multgenic cases where disease-causing variants in distinct genes can modulate the resulting phenotypic presentation (42, 43). The ability to assay a diverse set of genes in diseases with heterogeneous presentation could provide insight into disease mechanisms that had previously been unsolved.

There are limitations of long-read sequencing approaches, such as TaLon-SeqMD, compared with current short-read sequencing approaches, such as per-base accuracy and sample throughput. Furthermore, the lack of annotation of complex variants, such as SVs, limits the ability to assign pathogenicity and provide complete molecular diagnosis in cases. With continued development of long-read sequencing technology and improved annotation of SVs (44), long-read sequencing offers the promise to uncover precise molecular mechanisms of disease in genetic disorders. Moreover, there is mounting evidence that long reads offer distinct advantages in disease-variant discovery compared with standard short-read sequencing, especially for difficult-to-detect variant calls (45). In certain IRDs, such as one of the most common forms of X-linked RP caused by the *RPGR* gene (46), disease-causing variants can be refractory to analysis by short-read next-generation sequencing. Those with variants in the mutational hotspot of *ORF15* (46) have a more severe phenotype (47), but this region is highly repetitive, which makes it difficult to uniquely map with short-read sequencing. We find that even with reduced throughput runs compared with whole-genome short-read sequencing, we can unambiguously map reads to this region with long-reads, whereas there is poor read mapping with short-read genome sequencing (Supplemental Figure 6). With gene therapy trials aimed at treating this genotypic form of disease (48), our approach would better identify individuals who would benefit from intervention.

Overall, we demonstrate selective long-read sequencing of genomic regions of interest using a simple input configuration file on the ONT platform. This approach allows incredible flexibility in genomic coverage and panel development to generate rapid, phased data sets to better characterize Mendelian diseases. In this paper, we show this enables enhanced read depth to generate haplotype-phased reads from a single flow cell, which allows calling small variants in coding and noncoding regions as well as larger, more complex SVs to provide molecular diagnosis from the proband without the need for further familial sequencing. We anticipate that this approach will provide researchers and clinicians with a new paradigm to better resolve the genetic etiologies of Mendelian disorders and better guide clinical management of disease.

## Methods

### Sex as a biological variable.

Both male and female participants were enrolled in this research study.

### Blood sample collection and gDNA extraction of study patients.

Clinical diagnosis of IRD was based on history, ophthalmology, and audiology findings. A venipuncture blood of 2 mL was obtained from study individuals and gDNA was isolated using the MagAttract High Molecular Weight genomic DNA isolation kit (Qiagen). Deidentified individual samples underwent diagnostic testing in certified clinical laboratories for verification of variants identified from long-read sequencing.

### Short-read library preparation and sequencing, variant calling, and variant annotation.

Approximately 750 ng of gDNA was sheared using a Covaris LE220 focused ultrasonicator targeting 380-bp inserts and then subjected to a series of library construction steps utilizing the Roche KAPA Hyper Prep kit (KR0961 v1.14) and NovaSeq 6000 S4 Reagent Kit v1.5 (300 cycles) for short-read Illumina sequencing. Base calls were generated in real time on the Illumina NovaSeq 6000 instrument. BAM files were aligned to a human reference (GRCh38) using Burrows-Wheeler Aligner v0.7.15 (49). A pipeline based on the Genome Analysis Toolkit (GATK) (50) (v4.2.6.1) following the best practices was used (50, 51). The filtered BAM file was variant called using GATK HaplotypeCaller, and the output VCF underwent base quality score recalibration (BQSR) using GATK BaseRecalibrator. The recalibration tables were then used with GATK ApplyBQSR to recalibrate the base quality scores, and the recalibrated BAM file then underwent a second round of variant calling using GATK HaplotypeCaller. The resulting VCF files underwent several variant quality score recalibration steps using GATK VariantRecalibrator in both SNV and INDEL modes, with parameters tuned for whole-genome sequencing.

### Long-read library preparation and targeted panel sequencing.

For long-read library preparation, approximately 1200 ng of gDNA was used to make sequencing libraries using the ONT Ligation Sequencing Kit (SQK-LSK110), with slight modifications of the manufacturer’s protocol. As a modification to these instructions, 1.5 times the suggested amount of AMPure XP beads were used and 80% (instead of 70%) ethanol was used for the bead washing steps. During the adapter ligation and clean-up step, the Long Fragment Buffer was used to enrich DNA fragments greater than 3 kb in length. The resulting DNA library was loaded onto an R9.4.1 flow cell for sequencing on an ONT MinION Mk1B device. A GPU-accelerated version of guppy (v6.0.7, API version 10.1.0, ONT) was used for basecalling in real time using the “super-accurate” model parameters on a custom Linux-based computing workstation equipped with 2 NVIDIA RTX A6000 graphics cards and AMD Treadripper Pro 4995WX 64-core, 128-thread desktop processor. Target regions were enriched using Readfish (21) adaptive sampling technology implemented during real-time sequencing. To perform adaptive sampling for in silico enrichment, we prepared a BED file of each of our 373 IRD gene loci with a 50-kb buffer of each side. Sequencing experiments were run for up to 72 hours or until all the pores were inactive.

### Reference assembly refinement for proper alignment of reads to targeted genomic loci.

GRCh38 (23) was the reference genome used for targeting purposes using a BED file of the prespecified 373 genomic loci. A linear regression analysis was done to analyze the expected reads of each targeted genomic locus (calculated as a fraction of total bases of that locus divided by the total bases of all targeted bases) to the observed reads that uniquely mapped to each locus after a sequencing run. This analysis demonstrated that 31 genomic loci fell to the left of the regression line, indicative of a reduced number of observed reads from the sequencing run. The regions were then individually examined and we found that the reads that did map to each were denoted as supplementary reads because of duplicate regions of the genome. These duplicate regions of the genome were hard masked and the resulting linear regression analysis resulted in right shift of the discrepant points. The generated hard-masked GRCh38 genome reference file was used for subsequent alignment of all sequencing experiments.

### Sequence haplotagging, variant calling, and variant annotation of long-read data.

FASTQ files were generated using Dorado and aligned to the generated hard-masked GRCh38 assembly using minimap2 (52). The BAM file was collated, duplicates marked, and the reads filtered for a minimum alignment quality score of MAPQ 50 and secondary, supplementary, and optical duplicates were removed using SAMtools (https://github.com/samtools/samtools). Small variants (SNVs and indels) were called using PEPPER and haplotyping was achieved using Margin (https://github.com/kishwarshafin/pepper). SVs were analyzed with DeBreak (35). The DeepVariant pipeline was used to generate a phased variant call file (VCF) (53). The VCF files were then annotated with haplotype and phase-block information, variant depth, variant quality, variant effect predictor annotations, ClinVar clinical significance, allele frequency obtained from gnomAD (https://gnomad.broadinstitute.org/ Accessed August 31, 2024.), and Combined Annotation-Dependent Depletion (CADD) score to aid in analysis and prioritization of candidate variants.

### Long-read sequencing enrichment and phase breadth calculations.

Sequencing enrichment of targeted genomic loci was calculated by examining the average target genomic coverage from adaptive sampling and dividing that value by the whole-genome coverage generated by the nonadaptive reads across mappable regions of the genome. The average target genomic region coverage was assessed as total base pairs divided by the size of the sampled genomic region. This coverage was then averaged across all 373 targeted genomic loci. For whole-genome coverage analysis, the targeted genomic regions were excluded and the remaining genomic regions were assayed as a 10-kb sliding window to obtain average read depth across the human genome. Phase breadth was assessed as the percentage of a target genomic region that could be accurately phased. Whereas for each genomic loci, a 50-kb overhang of coding regions was used for targeting, the phase breadth was calculated for the strict boundaries of the start and stop of the coding regions of each targeted gene. Phase blocks were examined for each gene to assess the level of phased coverage and reported as a percentage of the total gene. This was done for all 373 genes in the panel for each sequencing run to assess phase breadth across all experiments.

### Splicing assay.

A minigene splicing assay was performed using a minigene split GFP construct (54), in which N- and C-terminal parts of the GFP gene were separated by *SMN1* introns 7 and 8 (NM_000344). Reference and mutated gene fragments (960 bp) flanked with 30-bp vector homology arms were synthesized (TWIST Bioscience) and cloned into the minigene construct (Gibson Assembly Master Mix, New England Biolabs). After Sanger sequencing verification of all constructs, they were transfected into HEK293 cells (Lipofectamine 3000, Thermo Fisher Scientific). Forty-eight hours after transfection, total RNA was extracted from the transfected cells (RNeasy Mini Kit, Qiagen) and cDNA was generated using random hexamer primers (SuperScript IV Synthesis Kit, Thermo Fisher Scientific). Subsequently, the minigene transcripts were amplified from the cDNA using primers specific for the split GFP fragments: Forward primer, 5′-CACACTGGTGACAACATTTACATAC-3′; Reverse primer, 5′-GAAATCGTGCTGTTTCATGTGATC-3′.

The PCR products were column purified (DNA Clean & Concentrator-5, Zymo Research) and analyzed with next-generation amplicon sequencing (MiSeq, Illumina, Ocular Genomics Institute Genomics Core). The splicing pattern analysis was performed by aligning the sequence reads to the human genome build 38 (Hg38) (STAR Aligner2; https://github.com/alexdobin/STAR) and visualizing the reads in the Integrated Genome Viewer.

### Statistics.

For each individual, per-base coverage and phase breadth of the targeted set of genomic loci from the sequencing run were calculated. A nonparametric box-and-whisker plot was used to display the median (line within the box) and lower and upper quantiles (bounds of the box), with bars showing the minimum and maximum values. To examine targeting of each genomic loci, Seaborn was run in Python (https://github.com/python) for drawing linear regression models. A standard linear regression was used to fit the log of the expected reads at each targeted locus to the log of the actual observed reads at each targeted locus.

### Study approval.

Study individuals were consented for genome sequencing under an approved protocol by the institutional review board at the University of Washington, Seattle, Washington (STUDY00014158). Written informed consent was obtained from all study individuals or parental guardians. Experiments were conducted according to the principles expressed in the Declaration of Helsinki.

### Data availability.

The genome variant data in this study are included within the published article. Genome sequencing data are not publicly available due to privacy and patient anonymity issues. Access to deidentified genome sequencing data will require an IRB-approved collaboration and Data Usage Agreement. Values for graphical representation of data presented in figures and supplemental figures are provided in the Supporting Data Values file.

## Author contributions

KN, RNVG, and DM designed the research. KN, JH, RS, and KMB performed the research. KN, RNVG, and DM contributed reagents/analytic tools. KN, JH, RS, EMP, KMB, MM, LAE, PY, JRC, RNVG, and DM recruited subjects and analyzed data. KN, RNVG, and DM wrote the manuscript.

## Supplementary Material

Supplemental data

Supporting data values

## Version 1 09/12/2024

In-Press Preview

## Version 2 10/22/2024

Electronic publication
