# Supplementary material for: Targeted long-read sequencing enriches disease-relevant genomic regions of interest to provide complete Mendelian disease diagnostics
Source: JCI Insight. 2024 Sep 12;9(20):e183902. doi: 10.1172/jci.insight.183902 (PMC11530123; doi:10.1172/jci.insight.183902)
Supplement: Supplemental data [file jciinsight-9-183902-s141.pdf]

Supplemental Data

Targeted long-read sequencing enriches disease-relevant genomic regions of interest to provide complete Mendelian disease diagnostics

Kenji Nakamichi[1,2], Jennifer Huey[1,2], Riccardo Sangermano[3], Emily M. Place[3], Kinga M. Bujakowska[3], Molly Marra[4], Lesley A. Everett[4], Paul Yang[4], Jennifer R. Chao[1,2], Russell N. Van Gelder[1,2,5], Debarshi Mustafi[1,2,6,7*]

[1]Department of Ophthalmology, University of Washington, Seattle, WA, 98109, [2]Roger and Karalis Johnson Retina Center, Seattle, WA, 98109, [3]Ocular Genomics Institute, Department of Ophthalmology, Massachusetts Eye and Ear, Harvard Medical School, Boston, Massachusetts 02115, [4]Casey Eye Institute, Oregon Health & Science University, Portland, Oregon 97239, [5]Departments of Laboratory Medicine and Pathology and Biological Structure, University of Washington, Seattle, WA, 98195, [6]Brotman Baty Institute for Precision Medicine, Seattle, WA 98195, [7]Division of Ophthalmology, Seattle Children's Hospital, Seattle, WA, 98105

***Corresponding Author:** Debarshi Mustafi

Department of Ophthalmology, University of Washington and [2]Roger and Karalis Johnson Retina Center, 750 Republican St, E273, Seattle, WA, 98109

Phone: (206) 221-2029

E-mail: debarshi@uw.edu

Supplemental Table 1. List of genes targeted using adaptive sampling for IRD panel coverage of each study subject

ABCA4, ABCC6, ABCD1, ABHD12, ACBD5, ACO2, ADAM9, ADAMTS18, ADAMTSL4, ADGRA3, ADGRV1, ADIPOR1, AGBL5, AHI1, AHR, AIPL1, ALMS1, ALPK1, AMACR, ARHGEF18, ARL13B, ARL2BP, ARL3, ARL6, ARMC9, ARR3, ARSG, ASRGL1, ATF6, ATOH7, B9D1, B9D2, BBIP1, BBS1, BBS10, BBS12, BBS2, BBS4, BBS5, BBS7, BBS9, BEST1, C12orf65, C1QTNF5, C21orf2, C2orf71, C5orf42, C8orf37, CA4, CABP4, CACNA1F, CACNA2D4, CAPN5, CC2D2A, CCT2, CDH23, CDH3, CDHR1, CEP104, CEP120, CEP164, CEP19, CEP250, CEP290, CEP41, CEP78, CEP83, CERKL, CFAP57, CFH, CHM, CIB2, CISD2, CLCC1, CLEC3B, CLN3, CLN5, CLN6, CLN8, CLRN1, CLUAP1, CNGA1, CNGA3, CNGB1, CNGB3, CNNM4, COL11A1, COL11A2, COL18A1, COL2A1, COL9A1, COL9A2, COL9A3, COQ2, CPE, CRB1, CRPPA, CRX, CSPP1, CTC1, CTNNA1, CTNNB1, CTSD, CWC27, CYP4V2, DFNB31, DHDDS, DHX32, DHX38, DNAJC17, DNAJC5, DRAM2, DSCAML1, DTHD1, DYNC2H1, DYNC2I2, EFEMP1, ELOVL4, EMC1, ERCC6, ESPN, EXOSC2, EYS, FAM161A, FBLN5, FDXR, FLVCR1, FRMD7, FSCN2, FZD4, GDF6, GDPD1, GJB6, GNAT1, GNAT2, GNB3, GNPTG, GNS, GPR143, GPR179, GPR45, GRK1, GRM6, GRN, GUCA1A, GUCA1B, GUCY2D, HARS, HCN1, HGSNAT, HK1, HMCN1, HMX1, IDH3A, IDH3B, IFT140, IFT172, IFT27, IFT43, IFT74, IFT80, IFT81, IFT88, IMPDH1, IMPG1, IMPG2, INPP5E, INVS, IQCB1, ITM2B, JAG1, KCNJ13, KCNV2, KIAA0556, KIAA0586, KIAA0753, KIAA1549, KIF11, KIF7, KIZ, KLHL7, LAMA1, LARGE1, LCA5, LRAT, LRIT3, LRMDA, LRP2, LRP5, LYST, LZTFL1, MAK, MAPKAPK3, MERTK, MFN2, MFRP, MFSD8, MIR204, MKKS, MKS1, MMACHC, MPDZ, MTPAP, MTTP, MVK, MYO7A, NAGLU, NBAS, NDP, NEK2, NEUROD1, NMNAT1, NPHP1, NPHP3, NPHP4, NR2E3, NR2F1, NRL, NXNL1, NYX, OAT, OCA2, OFD1, OPA1, OPA3, OPN1SW, OR2W3, OTX2, P3H2, PANK2, PAX2, PAX6, PCDH15, PCYT1A, PDE6A, PDE6B, PDE6C, PDE6D, PDE6G, PDE6H, PDSS1, PDSS2, PDZD7, PEX1, PEX10, PEX11B, PEX12, PEX13, PEX14, PEX16, PEX19, PEX2, PEX26, PEX3, PEX5, PEX6, PEX7, PGK1, PHYH, PISD, PITPNM3, PLA2G5, PLK4, PNPLA6, POC1B, POC5, POMGNT1, PPT1, PRCD, PRDM13, PRKCG, PROM1, PRPF3, PRPF31, PRPF4, PRPF6, PRPF8, PRPH2, PRPS1, RAB28, RAX2, RBP1, RBP3, RBP4, RCBTB1, RD3, RDH11, RDH12, RDH5, REEP6, RGR, RGS9, RGS9BP, RHO, RIMS1, RLBP1, ROM1, RP1, RP1L1, RP2, RP9, RPE65, RPGR, RPGRIP1, RPGRIP1L, RS1, RTN4IP1, SAG, SAMD11, SCAPER, SCLT1, SDCCAG8, SEMA4A, SGSH, SIX6, SLC24A1, SLC24A5, SLC25A46, SLC45A2, SLC7A14, SNRNP200, SPATA7, SPP2, SRD5A3, TCTN1, TCTN2, TCTN3, TEAD1, TIMM8A, TIMP3, TMED7, TMEM107, TMEM126A, TMEM138, TMEM216, TMEM231, TMEM237, TMEM67, TOPORS, TPP1, TRAF3IP1, TREX1, TRIM32, TRNT1, TRPM1, TSPAN12, TTC21B, TTC8, TTLL5, TTPA, TUB, TUBB4B, TUBGCP4, TUBGCP6, TULP1, TYR, TYRP1, UNC119, USH1C, USH1G, USH2A, VCAN, VPS13B, VSX2, WDPCP, WDR19, WDR35, WFS1, YME1L1, YPEL2, ZNF408, ZNF423, ZNF513

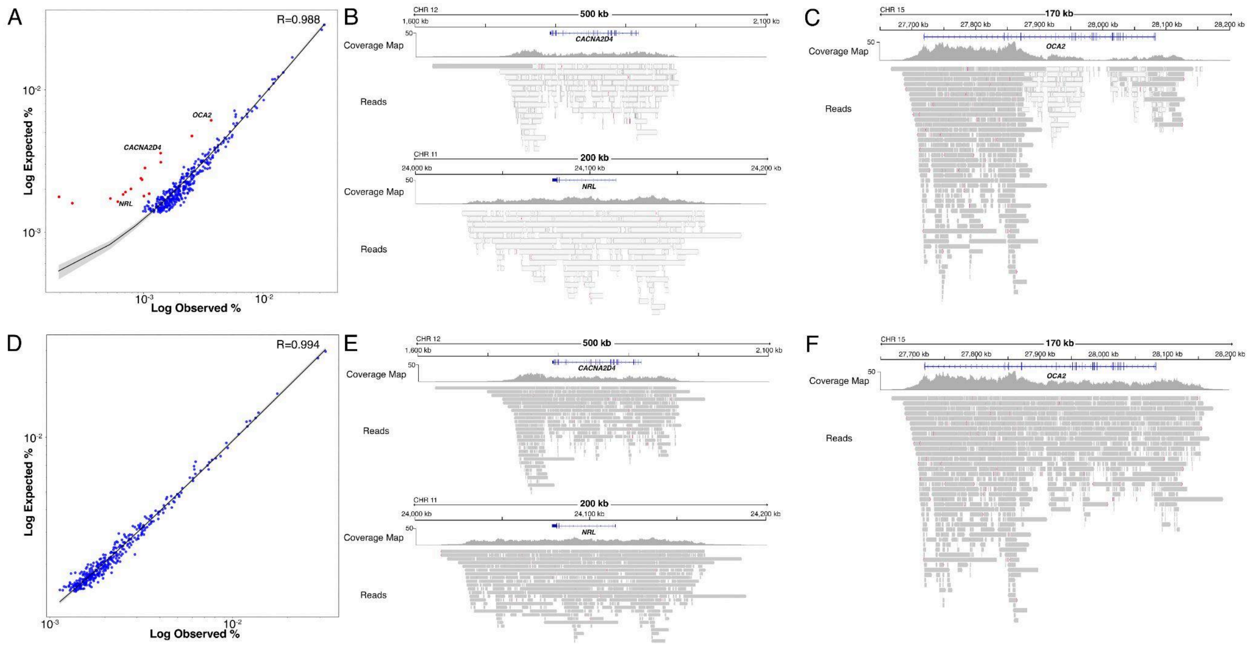

Supplemental Figure 1. Masking of genomic regions of the current build provides on-target coverage of every gene in our panel. (A) Initial run of our panel showed that the majority of genes had excellent correlation of expected and observed coverage, but a subset of genes were not being properly targeted. The reason for that is some of the genes such as **(B)** *CACNA2D* and *NRL* were not being properly targeted due to duplicated regions in the genome that resulted in improper alignments of the entire gene or **(C)** in the cases of *OCA2*, improper targeting of a specific portion of the gene. **(D)** By masking these regions all the genes had excellent correlation of expected and observed coverage as evidenced by the resulting alignments of **(E)** *CACNA2D*, *NRL* and **(F)** *OCA2*.

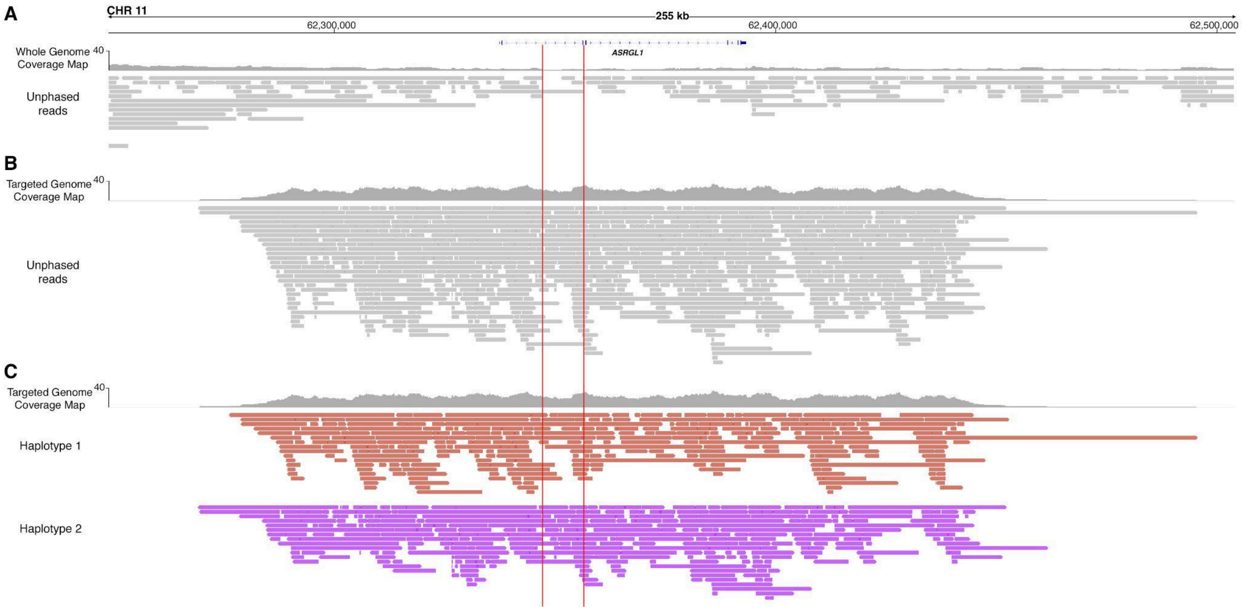

Supplemental Figure 2. TaLon-SeqMD provides more uniform coverage in regions of interest compared to whole genome sequencing. (A) When examining targeted regions of interest there are gaps in coverage with whole genome sequencing whereas **(B)** targeted sequencing focused on these regions provide not only greater depth of coverage but also more uniform coverage, which allows for **(C)** phasing of long-read data.

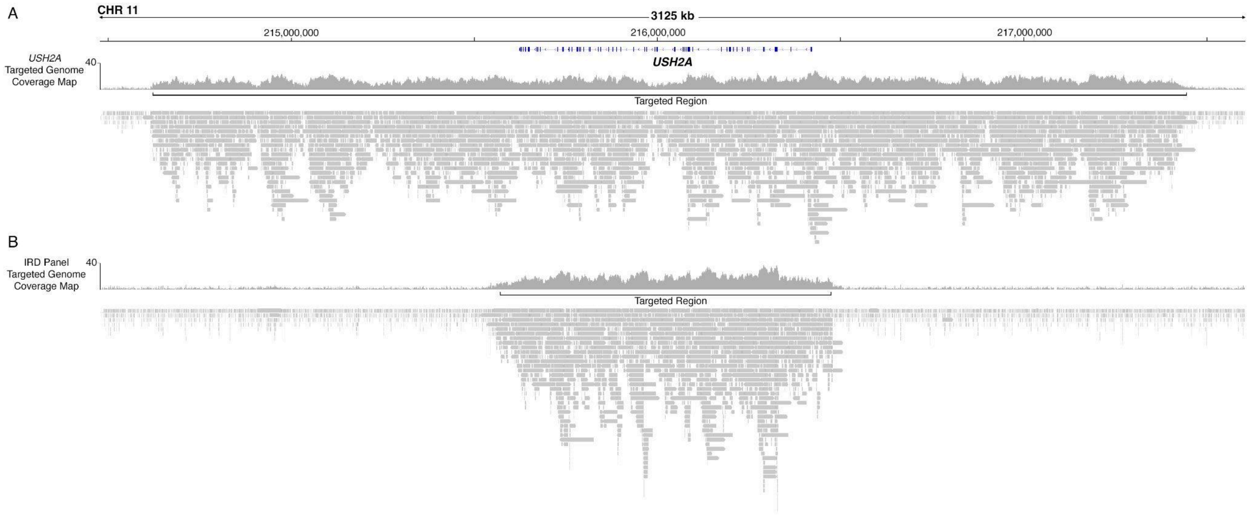

Supplemental Figure 3. Expansion of genome targeting from a single gene to multiple genomic loci does not diminish per-base coverage of regions of interest. When examining the *USH2A* locus, coverage from **(A)** single-gene targeting and **(B)** panel targeting did not produce any difference in total coverage of the pre-targeted regions. In the single-gene experiment, a 1 megabase flanking region was targeted on either side of the *USH2A* gene locus compared to a 50 kilobase flanking region that was targeted on either side of the *USH2A* gene locus for panel sequencing.

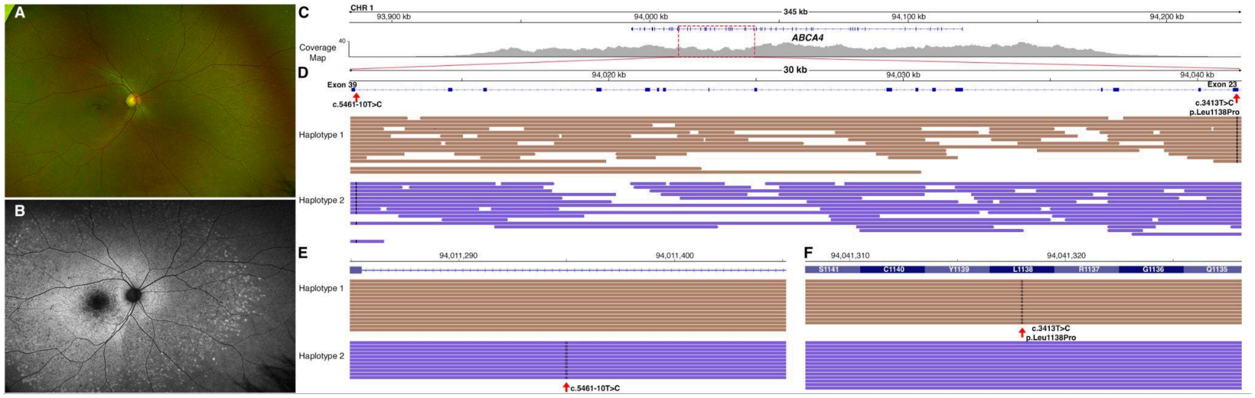

Supplemental Figure 4. Haplotype-resolved reads allow for VUS reclassification in a case with *ABCA4*-related Stargardt disease. (A) Fundus photo of the right eye with retinal flecks and **(B)** fundus autofluorescence photo of the right eye with hyperautofluorescent flecks and hypoautofluorescence signal in the macula were consistent with a clinical phenotype juvenile fleck macular dystrophy such as Stargardt disease. **(C)** Long-read sequencing provided full coverage of the *ABCA4* gene locus and **(D)** closer examination of the 30 kb region encompassing exons 23 to 39 showed that the **(E)** a known pathogenic variant (c.5461-10T>C) was in trans to a **(F)** VUS, c.3413T>A (p.Leu1138Pro) in exon 23 was in *trans*. Further examination of the VUS revealed there were two other missense variants at this codon that are classified as likely pathogenic (p.Leu1138His and p.Leu1138Phe). The *ABCA4* c.3413T>A variant is also rare in controls, *in silico* tools predict this variant impacts protein function, and this variant has a CADD score of 29.1. Thus, the *ABCA4* c.3413T>A variant can be reclassified as likely pathogenic based on ACMG guidelines.

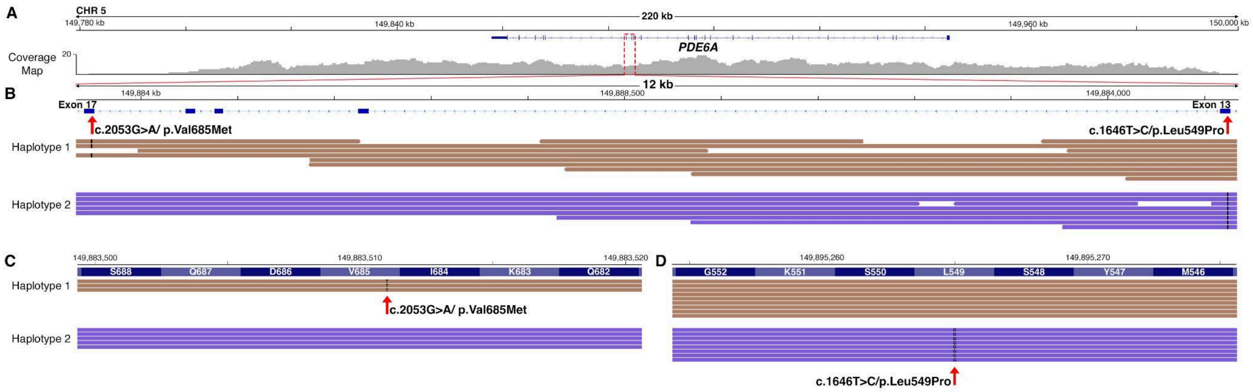

Supplemental Figure 5. Haplotype-resolved reads allow for VUS reclassification in a case with *PDE6A*-related rod-cone dystrophy. (A) Long-read sequencing provided full coverage of the *PDE6A* gene locus and **(B)** closer examination of the 12 kb region encompassing exons 13 to 17 showed that the **(C)** a known pathogenic variant (c.2053G>A, p.Val685Met) was in trans to a **(D)** VUS, c.1646T>C (p.Leu549Pro), in exon 13 was in *trans*. Further examination of the VUS revealed that this variant is rare in large population databases, *in silico* tools predict this variant has a deleterious impact on protein function, and this variant has a CADD score of 25.1. Furthermore, this variant is at a locus that is highly conserved among mammals. Thus, the *PDE6A* c.1646T>C (p.Leu549Pro) can be reclassified as likely pathogenic based on ACMG guidelines.

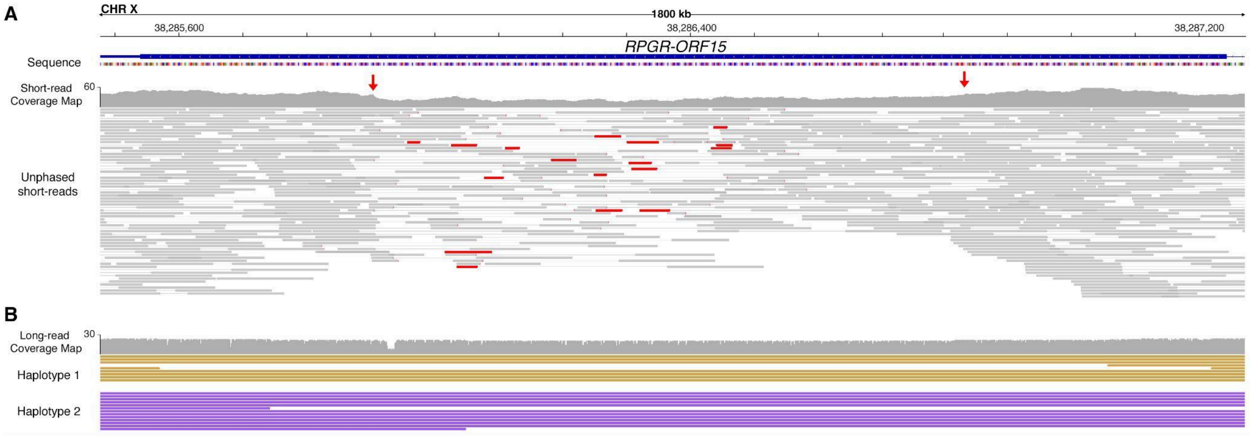

Supplemental Figure 6. Long-reads allow for accurate mapping in complex disease-relevant regions of the genome that are not properly resolved with short-reads. The ORF15 region of *RPGR* is a hotspot for disease-variants, but the repetitive nature of bases in the region makes it difficult to accurately map short-reads. **(A)** In the region denoted by red arrows, short-reads fail quality control are shown in red and thus cannot be accurately mapped with its mate pairs. **(B)** In contrast with long-reads the region is fully mapped and because this subject was female, we can appreciate haplotype-resolved reads encompassing the entire region. Most importantly, despite a 2-fold higher number of reads in this region with short-reads, the region can still not be accurately mapped, demonstrating that read length, not read depth, is important in these medically complex regions of the genome.
